# Arginine vasopressin in the medial amygdala causes greater post-stress recruitment of hypothalamic vasopressin neurons

**DOI:** 10.1186/s13041-021-00850-2

**Published:** 2021-09-15

**Authors:** Wen Han Tong, Samira Abdulai-Saiku, Ajai Vyas

**Affiliations:** 1 School of Biological Sciences, Nanyang Technological University, 60 Nanyang Drive, Singapore, 637551 Singapore; 2 Department of Neurology and Weill Institute for Neurosciences, University of California, San Francisco, CA 94158 USA

**Keywords:** Defensive behaviors, Extended amygdala, Innate fear, Nonapeptides, Paraventricular hypothalamus, Testosterone

## Abstract

Arginine vasopressin (AVP) is expressed in both hypothalamic and extra-hypothalamic neurons. The expression and role of AVP exhibit remarkable divergence between these two neuronal populations. Polysynaptic pathways enable these neuronal groups to regulate each other. AVP neurons in the paraventricular nucleus of the hypothalamus increase the production of adrenal stress hormones by stimulating the hypothalamic–pituitary–adrenal axis. Outside the hypothalamus, the medial amygdala also contains robust amounts of AVP. Contrary to the hypothalamic counterpart, the expression of extra-hypothalamic medial amygdala AVP is sexually dimorphic, in that it is preferentially transcribed in males in response to the continual presence of testosterone. Male gonadal hormones typically generate a negative feedback on the neuroendocrine stress axis. Here, we investigated whether testosterone-responsive medial amygdala AVP neurons provide negative feedback to hypothalamic AVP, thereby providing a feedback loop to suppress stress endocrine response during periods of high testosterone secretion. Contrary to our expectation, we found that AVP overexpression within the posterodorsal medial amygdala increased the recruitment of hypothalamic AVP neurons during stress, without affecting the total number of AVP neurons or the number of recently activated neurons following stress. These observations suggest that the effects of testosterone on extra-hypothalamic AVP facilitate stress responsiveness through permissive influence on the recruitment of hypothalamic AVP neurons.

**Supplementary Information:**

The online version contains supplementary material available at 10.1186/s13041-021-00850-2.

Continual interactions between the brain and endocrine system are required for appropriate response to environmental variability; a particularly robust example of this interaction is the reciprocity between stress and reproduction being a particularly robust example. Gonadal hormones typically suppress the stress endocrine response through negative feedback originating in the brain. Similarly, stress hormones from the adrenal glands suppress the reproductive hormones through both central and peripheral mechanisms. Thus, stress and reproduction can be conceptualized as two mutually exclusive neuroendocrine landscapes that allow an individual to exist in two separate behavioral states depending on the internal metabolic milieu and external environmental conditions.

Testosterone, a male gonadal hormone, easily crosses the blood–brain barrier. It plays a role in regulating arginine vasopressin (AVP) transcription and hypothalamic–pituitary–adrenal (HPA) activity by acting through androgen receptors [1]. Various sites in the brain express receptors for testosterone or its metabolites. Among these, the medial amygdala (MeA) is especially interesting because it contains a sexually dimorphic population of extra-hypothalamic AVP neurons [2]. AVP expression in this population requires the continual presence of androgens during adulthood [3]. Interestingly, the bed nucleus of the stria terminalis (BNST) shares characteristics identical to the MeA in steroid responsiveness, cytoarchitecture, and neurochemistry, and its AVP system is also dependent on circulating gonadal steroids [4]. The AVP system of both MeA and BNST is sexually dimorphic in rats and mice, although more AVP neurons are present in males [5]. The MeA also sends copious monosynaptic and polysynaptic projections to the paraventricular nucleus of the hypothalamus (PVN); this constitutes a potential pathway for testosterone to affect the stress endocrine axis through AVP production in the MeA and its downstream influence on the PVN [6]. The PVN also contains AVP neurons that are functionally distinct from MeA-AVP neurons. Unlike MeA-AVP transcription, PVN-AVP transcription is not directly regulated by testosterone. PVN-AVP neurons are recruited during stress exposure and potentiate the ability of corticotropin-releasing hormone to release adrenocorticotropic hormones from the pituitary [7]. Moreover, testosterone exerts an inhibitory effect on HPA effector neurons by negatively modulating the transcription of the AVP gene in hypophysiotropic PVN neurons [8, 9]. The removal of gonads during pre-weaning periods increases the recruitment of PVN-AVP neurons and increases the responsiveness of the stress endocrine axis to environmental stressors [10]. Thus, extra-hypothalamic MeA-AVP and hypothalamic PVN-AVP are anatomically connected. However, these populations seem to be paralogous in their relationship with the reproductive and stress endocrine axes.

With this background information, we aimed to investigate whether an experimental manipulation that increases AVP expression within the posterodorsal MeA causes the reciprocal attenuation of PVN-AVP recruitment during stress.

Mice expressing Cre recombinase from AVP promoter (Cre+) and controls (Cre−) were infused with a viral vector in the posterodorsal MeA, delivering the AVP gene requiring recombination for its expression from a robust promotor (Additional file 1: Fig. S1; Additional file 2: Additional Materials and Methods). After 4-weeks, the mice were exposed to bobcat urine for 20 min, and sacrificed after 90 min. Coronal sections spanning the PVN were stained using antibodies for AVP and Fos, which is a marker for the recent neuronal activity. Neurons positive for AVP and/or Fos were counted relative to the total DAPI counts (Fig. 1a). The cohort of animals used in this study was earlier analyzed in another study with respect to MeA neurons [11].

**Fig. 1:**
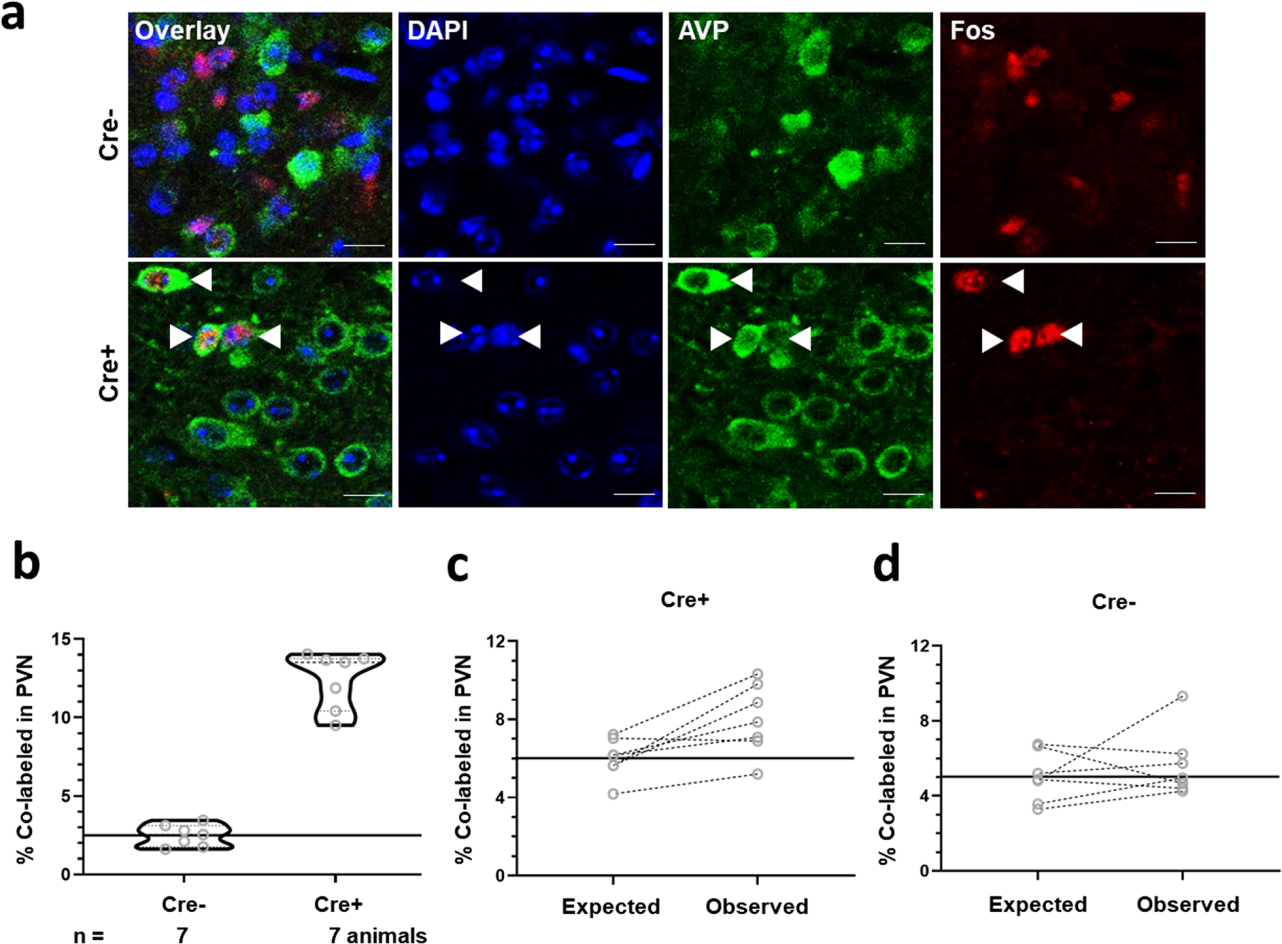
Representative images showing immunofluorescence for arginine vasopressin (AVP), Fos (a marker for recent neuronal activity), and nuclear boundaries (DAPI) within paraventricular hypothalamus (**a**). Animals were surgically infused with viral vectors containing AVP within the posterodorsal medial amygdala. The expression of cargos was dependent on the availability of Cre recombinase in the genome of animals. Examples from Cre− and Cre+ are depicted with the scale bar of 10 μm. Effects of experimental manipulation on the paraventricular hypothalamus neurons co-labeled with AVP and Fos in response to cat odor exposure (**b**). The number of co-labeled neurons relative to the total number of neurons is depicted on the ordinate. Violin plot depicts the median and inter-quartile range along with the raw values for all data points (n underneath the abscissa). Solid lines parallel to abscissa represent the mean for the control group. Stochastic expectation and experimental observation of co-labeled neurons in the treated (Cre+, **c**) and control (Cre−, **d**) groups. The raw values for each individual animal are depicted. Solid lines parallel to the abscissa represent mean expectation for that group

AVP overexpression within the MeA did not affect the number of PVN neurons expressing AVP (Additional file 1: Fig. S2a; t_12_ = 0.26, *p* = 0.798; ∆x̅ = 1.2 ± 4.5%). Similarly, AVP overexpression within the MeA did not cause a significant effect on PVN neurons showing recent activation in response to bobcat odor exposure (Additional file 1: Fig. S2b; t_12_ = 1.8, *p* = 0.097; ∆x̅ = 3.0 ± 1.6%). Thus, AVP overexpression within the MeA did not have a robust effect on the number of PVN AVP- or Fos-positive neurons. 

We then compared the number of co-labeled PVN neurons, specifically quantifying those neurons that express AVP and have been active (Fos-positive) during recent exposure to bobcat odor. MeA-AVP overexpression robustly increased the number of co-labeled PVN-AVP neurons (t_12_ = 13.45, *p* < 0.001; ∆x̅ = 9.9 ± 0.7%). All animals with the Cre+  genotype exhibited more co-labeling than the maxima of Cre− littermates (Fig. 1b; Hedges' g = 6.5). The effect size of this comparison was well above the conventional frame of reference that defines the effect sizes of > 0.8 as being “large”. Nearly one-third of AVP+ neurons (31.8% ± 1.6%) in the PVN exhibited Fos expression in the Cre+ group compared with 6.1% (± 1.8) of AVP+ neurons in the respective controls (t_12_ = 15.2, *p* < 0.001; ∆x̅ = 25.6% ± 1.7%). To analyze whether the number of co-labeled neurons reflected the stochastic chance of encountering a Fos-positive and AVP-positive neuron, we calculated the expected frequency of co-labeling for each animal as the mathematical product of AVP and Fos frequencies. Expected frequency was then compared with the observed frequency of co-labeling by using paired t-test. Animals in the Cre+ group were found to exhibit co-labeling that was significantly higher than the stochastic expectation (Fig. 1c; t_6_ = 3.4, *p* = 0.014). In contrast, the expected and observed co-labeling frequencies did not differ significantly in the Cre− group (Fig. 1d; t_6_ = 0.8, *p* > 0.4). Thus, AVP overexpression within the MeA caused a greater-than-chance increase in the recruitment of PVN-AVP neurons after acute exposure to predator odor, without significantly affecting the total number of PVN neurons expressing AVP or those showing recent neuronal activity.

These results suggest that MeA-AVP neurons increase the activation of hypothalamic PVN-AVP neurons upon predator odor exposure. This preferential recruitment is evidenced by the extent of co-labeling that surpasses the probabilities calculated on the basis of the individual expected and observed frequencies of AVP- and Fos-labeled cells. MeA-AVP neurons play an important role in modulating the HPA axis and fear responses [11, 12]. AVP has also been suggested to promote stress responses in adult rats [6]. AVP expression in the MeA is maintained by circulating testosterone levels, whereas testosterone exerts an inhibitory effect on the HPA activity [3, 8]. Therefore, we postulate that MeA-AVP neurons may impose the same attenuating effect on the activation of PVN-AVP neurons. However, contrary to our expectation, increased AVP expression in MeA neurons that express AVP endogenously resulted in an upregulation in the recruitment of PVN-AVP neurons during acute stress.

Despite the absence of robust monosynaptic efferents to the PVN, the MeA sends and receives copious connections from the BNST [13]. GABAergic neurons from the BNST, in turn, send direct efferents to inhibit PVN neurons [14]. Thus, AVP upregulation in MeA-AVP neurons can potentially influence PVN neurons through the polysynaptic pathway, which comprises inhibitory neurons in the BNST. Upon stressor application, these GABAergic neurons may exert a disinhibition effect on PVN activity, resulting in the activation of PVN-AVP neurons. Another possibility is that MeA-AVP perturbation causes endocrine changes, resulting in the plasticity of PVN-AVP neurons. MeA-AVP neurons are hypothesized to be involved in sexual and affiliative behaviors that correspond to an increase in the production of gonadal testosterone in males; for example, exposure to estrous females recruits MeA neurons through converging olfactory inputs and, in parallel, corresponds to increased synthesis of testosterone [15]. Thus, genetic perturbation of MeA-AVP neurons can plausibly result in higher testosterone production by increasing the tonicity of the gonadal steroidogenesis. Interestingly, neurons in the BNST contain androgen receptors and respond to their activation by increasing AVP transcription in the PVN and post-stress recruitment of PVN-AVP neurons [16].

In conclusion, we provide evidence that the experimental overexpression of a nonapeptide neuronal population, known to have a role in the stress and reproductive endocrine axes, can concomitantly upregulate the activation of hypothalamic vasopressin neurons. GABAergic neuronal population of the BNST is a potential polysynaptic pathway that is responsible for such hyperactivation. Endocrine changes involving testosterone is another possibility behind the increased activation of PVN-AVP neurons upon perturbation of MeA-AVP neurons.

## Supplementary Information

**Additional file 1: Fig. S1.** Representative images showing immunofluorescence for arginine vasopressin (AVP), and nuclear boundaries (DAPI) within the posterodorsal medial amygdala. Animals were surgically infused with viral vectors containing AVP within the posterodorsal medial amygdala to induce AVP overexpression. The expression of cargos was dependent on the availability of Cre recombinase in the genome of animals. Examples from Cre− and Cre+ are depicted with the scale bar of 10 μm. **Fig. S2.** Effects of experimental manipulation on paraventricular hypothalamus arginine vasopressin (AVP, a) neurons and those expressing the immediate-early gene (Fos, b). The number of positive neurons relative to the total number of neurons is depicted on the ordinate of panels a and b. Violin plots in these panels depict the median and inter-quartile range along with the raw values for all data points (n underneath the abscissa). Solid lines parallel to the abscissa in panel a and b represent the mean for the corresponding control.

**Additional file 2:** Additional materials and methods.

## Data Availability

Authors confirm that, should the manuscript be accepted, the data supporting the results will be archived in an appropriate public repository such as Dryad or Figshare, and the data DOI will be provided at the end of the article.

## Abbreviations

AVP: Arginine vasopressin
BNST: Bed nucleus of the stria terminalis
HPA: Hypothalamic–pituitary–adrenal
MeA: Medial amygdala
PVN: Paraventricular nucleus of the hypothalamus
