# Supplementary figures and images for: Arginine vasopressin in the medial amygdala causes greater post-stress recruitment of hypothalamic vasopressin neurons
Source: Mol Brain. 2021 Sep 15;14:141. doi: 10.1186/s13041-021-00850-2 (PMC8442369; doi:10.1186/s13041-021-00850-2)

## Slide 1
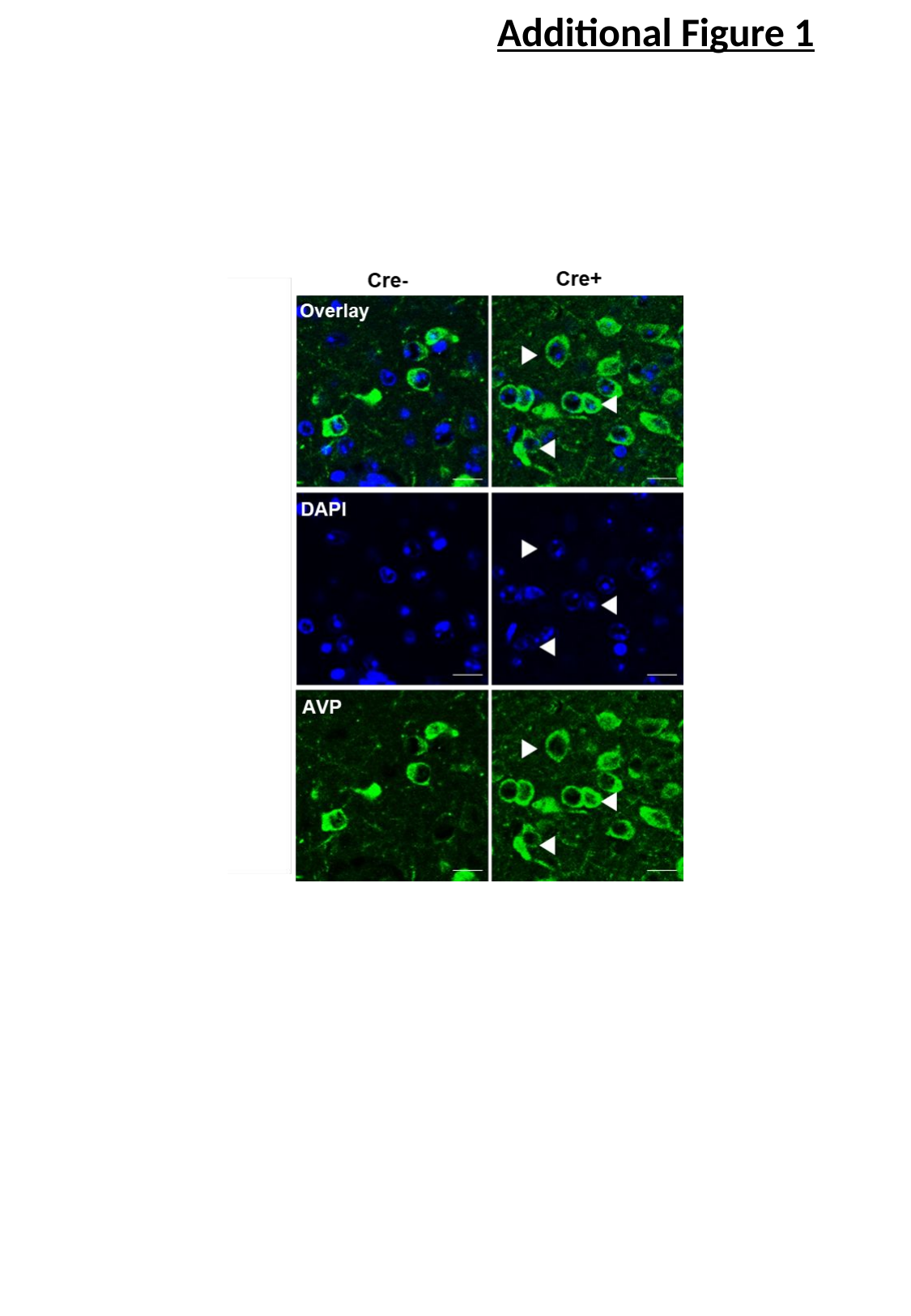

Additional Figure 1

## Slide 2
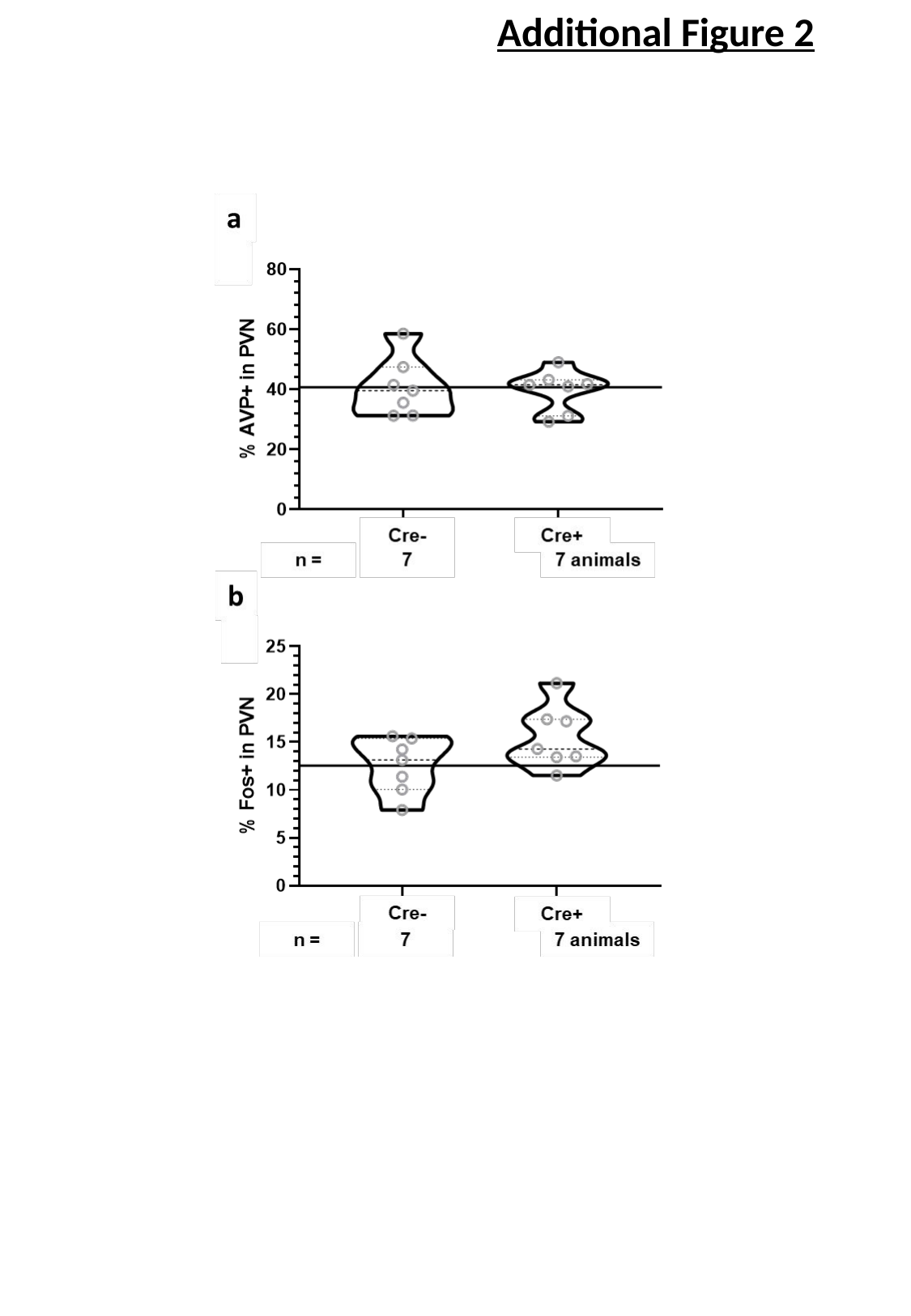

Additional Figure 2

Supplement: Supplementary file 1 — Additional file 1: Fig. S1. Representative images showing immunofluorescence for arginine vasopressin (AVP), and nuclear boundaries (DAPI) within the posterodorsal medial amygdala. Animals were surgically infused with viral vectors containing AVP within the posterodorsal medial amygdala to induce AVP overexpression. The expression of cargos was dependent on the availability of Cre recombinase in the genome of animals. Examples from Cre− and Cre+ are depicted with the scale bar of 10 μm. Fig. S2. Effects of experimental manipulation on paraventricular hypothalamus arginine vasopressin (AVP, a) neurons and those expressing the immediate-early gene (Fos, b). The number of positive neurons relative to the total number of neurons is depicted on the ordinate of panels a and b. Violin plots in these panels depict the median and inter-quartile range along with the raw values for all data points (n underneath the abscissa). Solid lines parallel to the abscissa in panel a and b represent the mean for the corresponding control. [file 13041_2021_850_MOESM1_ESM.pptx]
