# Supplementary material for: Arginine vasopressin in the medial amygdala causes greater post-stress recruitment of hypothalamic vasopressin neurons
Source: Mol Brain. 2021 Sep 15;14:141. doi: 10.1186/s13041-021-00850-2 (PMC8442369; doi:10.1186/s13041-021-00850-2)
Supplement: Supplementary file 2 — Additional file 2: Additional materials and methods. [file 13041_2021_850_MOESM2_ESM.docx]

**Additional File 2 - Additional Materials and Methods**

**Arginine vasopressin in the medial amygdala causes greater post-stress recruitment of hypothalamic vasopressin neurons.**

Wen Han Tong ^1^ *, Samira Abdulai-Saiku ^2^, and Ajai Vyas ^1^

^1^ School of Biological Sciences, Nanyang Technological University, 60 Nanyang Drive,

Singapore – 637551.

^2^ Department of Neurology and Weill Institute for Neurosciences, University of California, San Francisco, CA 94158, USA.

*Correspondence to:

Wen Han Tong

School of Biological Sciences, Nanyang Technological University,

60 Nanyang Drive, Singapore – 637551.

Phone: +65 6316 7088.

Email: wenhan.tong@ntu.edu.sg

**Materials and Methods**

**We acquired a transgenic line of C57BL/6J mice that expresses Cre recombinase in control of an AVP promotor (J**AX **stock #023530; Jackson Laboratory). Mice heterozygous for this mutation were bred with homozygous null mice, resulting in litters with a Mendelian mixture of Cre+ mutants and Cre- littermate controls. At seven week of age, all offspring were infused with an adeno-associated viral (AAV) vector delivering AVP gene**. This type 2 AAV vector contained a **floxed AVP gene that was downstream to a robust EF1α promoter and double inverted loxP elements, enabling overexpression of AVP in presence of Cre recombinase. Viral particles were packaged by Cyagen Biosciences at a concentration of >10^^11^ viral particles/mL. The viral vector was surgically delivered at posterodorsal MeA (**anteroposterior: –1.70, mediolateral: ± 2.00, and dorsoventral: –5.00 from bregma**; 10^^9^ viral particles per hemisphere). In approximately half of littermates that contained Cre, this treatment resulted in overexpression of AVP in those MeA neurons that endogenously express AVP. Other half of littermates served as corresponding controls (****Additional file 1: Figure 1). Animals were not genotyped till the end of the analysis; thus, experimenters were blind to the treatment.**

**Four weeks after the infusion of viral vectors, mice were habituated to a 46 x 9 cm rectangular arena for 3 consecutive days for 20 minutes sans bobcat urine. A novel odor (vanilla extract: 2 mL, 1:4 dilution) was exposed to the mice on the third day of habituation. On the fourth day, animals were acutely stressed by exposure to 1 mL of undiluted bobcat urine** (100% pure PredatorPee® bobcat urine from PredatorPee) **for 20 minutes in the arena. Animals were sacrificed after 1.5 hours through transcardial perfusion of buffered saline and 4% paraformaldehyde solution. Coronal brain sections containing PVN were stained with antibodies against AVP and an immediate early-gene marker for recent neuronal activity, *i.e.* Fos. Free-floating sections were incubated in a cocktail of primary antibodies for 72 hours at 4°C (guinea pig anti-AVP,#T-5048, Peninsula Laboratories, 1:1500; rabbit anti-Fos, #ABE457, Merck Millipore, 1:10000), followed by secondary antibodies for two hours (Alexa Fluor 488 AffiniPure donkey anti-guinea pig, #706-545-148, Jackson Immunoresearch Laboratories, 1:1000; Dylight 549 goat anti-rabbit, #DI-5149, Vector Laboratories, 1:1000). Sections were mounted using Gold Antifade Mountant with DAPI (#P36931, Life Technologies) to label nuclear boundaries. Images were captured for PVN neurons with the Carl Zeiss LSM 710 laser scanning confocal microscope using a 40X objective lens with a 1.2X digital magnification. Sections were optically sliced at 1 μm interval. Neurons from medial parvicellular part of paraventricular hypothalamic nucleus were imaged. All neurons showing positive immunoreactivity for AVP, or Fos, or colabeled neurons with both AVP and Fos were counted, relative to total DAPI positive neurons quantified and expressed as a percentage (Figure 1A). Statistical analysis was carried out using GraphPad Prism 7.0 software. Orthogonal planned comparisons were conducted between groups using Student's t-test. The magnitude of inter-group differences was computed using Hedges’ unbiased g.**

**The cohort of animals used here were earlier analyzed with respect to medial amygdala neurons [1].**

Reference

1. Tong, W.H., S. Abdulai-Saiku, and A. Vyas, *Medial amygdala arginine vasopressin neurons regulate innate aversion to cat odors in male mice.* Neuroendocrinology, 2020.
